# Mechanical Loading in Osteocytes Induces Formation of a Src/Pyk2/MBD2 Complex That Suppresses Anabolic Gene Expression

**DOI:** 10.1371/journal.pone.0097942

**Published:** 2014-05-19

**Authors:** Julia M. Hum, Richard N. Day, Joseph P. Bidwell, Yingxiao Wang, Fredrick M. Pavalko

**Affiliations:** 1 Department of Cellular and Integrative Physiology, Indiana University School of Medicine, Indianapolis, Indiana, United States of America; 2 Department of Anatomy and Cell Biology, Indiana University School of Medicine, Indianapolis, Indiana, United States of America; 3 School of Engineering, University of California San Diego, San Diego, California, United States of America; The University of Akron, United States of America

## Abstract

Mechanical stimulation of the skeleton promotes bone gain and suppresses bone loss, ultimately resulting in improved bone strength and fracture resistance. The molecular mechanisms directing anabolic and/or anti-catabolic actions on the skeleton during loading are not fully understood. Identifying molecular mechanisms of mechanotransduction (MTD) signaling cascades could identify new therapeutic targets. Most research into MTD mechanisms is typically focused on understanding the signaling pathways that stimulate new bone formation in response to load. However, we investigated the structural, signaling and transcriptional molecules that suppress the stimulatory effects of loading. The high bone mass phenotype of mice with global deletion of either Pyk2 or Src suggests a role for these tyrosine kinases in repression of bone formation. We used fluid shear stress as a MTD stimulus to identify a novel Pyk2/Src-mediated MTD pathway that represses mechanically-induced bone formation. Our results suggest Pyk2 and Src function as molecular switches that inhibit MTD in our mechanically stimulated osteocyte culture experiments. Once activated by oscillatory fluid shear stress (OFSS), Pyk2 and Src translocate to and accumulate in the nucleus, where they associate with a protein involved in DNA methylation and the interpretation of DNA methylation patterns –methyl-CpG-binding domain protein 2 (MBD2). OFSS-induced Cox-2 and osteopontin expression was enhanced in Pyk2 KO osteoblasts, while inhibition of Src enhanced osteocalcin expression in response to OFSS. We found that Src kinase activity increased in the nucleus of osteocytes in response to OFSS and an interaction activated between Src (Y418) and Pyk2 (Y402) increased in response to OFSS. Thus, as a mechanism to prevent an over-reaction to physical stimulation, mechanical loading may induce the formation of a Src/Pyk2/MBD2 complex in the nucleus that functions to suppress anabolic gene expression.

## Introduction

Pyk2 plays an important role in bone remodeling. [1]–[5]. Pyk2 null mice exhibit increased bone mass. [1], [6]. Reports differ on the explanation of the osteopetrotic phenotype of the Pyk2 knockout mouse. Once group reports the phenotype results from defective osteoclast function implicating Pyk2’s role in osteclast driven bone resporption, while another group contends increased osteoblast differentiation contributes to the osteopetrosis [7], [8]. Pyk2’s more well-known family member, FAK, serves as an important positive regulator of mechanical stimuli in osteoblasts [9]. Pyk2’s role in mediating the response of bone cells to mechanotransduction is unknown, but is suggested to be different than FAK’s [10]. Additionally, Src phosphorylates both FAK and Pyk2, while FAK and Pyk2 also associate and phosphorylate Src [11]–[14].

Similar to Pyk2, global disruption of Src, non-receptor tyrosine kinase, resulted in a mouse with a high bone mass phenotype, demonstrating the importance of Src in osteoblastogenesis. The function of both osteoclasts and osteoblasts is altered in Src−/− mice [15]–[17]. Osteoclast numbers are increased at the bone surface, but lack a ruffled border and are inactive [18]–[20]. Accelerated osteoblastogenesis was observed in the Src-null mice, suggesting Src activity plays a suppressive role in osteoblast differentiation [16]. These findings led to studies focused on producing a Src inhibitor to treat osteoporosis [21]–[24].

If both Pyk2 and Src function to balance bone mass by suppressing anabolic bone genes, it is also likely to affect the response of bone to mechanical loading. In the healthy mammalian skeleton, this process is mediated by osteocytes and osteoblasts that coordinate an appropriate response to mechanical loading resulting in localized net bone gain or loss depending on the type of load experienced at specific sites [25]–[27]. Osteocytes and osteoblasts sense and react to mechanical loads generated by the fluid flow through the canalicular system within bone [28]–[30]. *In vitro*, osteoblasts and osteocytes respond to mechanical load simulated via the application of oscillatory fluid shear stress (OFSS). Fluid shear stress causes distortions of the membranes of osteoblasts and osteocytes resulting in the enhanced expression of genes associated with osteoblast activity, up regulating cell proliferation and increasing the release of paracrine factors required for bones to elicit an anabolic response [31]–[33]. Both Pyk2 and Src increase their activation in response to OFSS [34]–[36].

We tested the hypothesis that Pyk2 and Src suppress anabolic targets of OFSS-induced MTD in osteoblasts and osteocytes. In this study we sought to determine Pyk2 and Src’s localization, effect on anabolic protein expression and gene transcription, and association with other signaling molecules under static and OFSS conditions.

## Methods

### Cell Culture Conditions

MC3T3 osteoblasts were obtained from ATCC, mouse calvarial osteoblasts (MCOB) were derived from WT neonatal mouse pups, as previously described [37], and Pyk2−/− osteoblasts were derived from Pyk2−/− mice as previously described [10]. Osteoblasts were cultured in minimal essential media alpha (MEM-α, Gibco, Life Technologies, Carlsbad, CA) containing 10% fetal calf serum (FCS) and 1% penicillin/streptomycin (Gibco, Life Technologies, Grand Island, NY). MLO-Y4 osteocytes were obtained from Dr. Linda Bonewald, UMKC and were cultured on collagen-coated plates (rat tail collagen type I, BD Biosciences, San Jose, CA) in MEM-α (Gibco, Life Technologies, Carlsbad, CA) supplemented with 5% FCS, 5% fetal bovine serum (FBS) and 1% penicillin/streptomycin (Gibco, Life Technologies, Grand Island, NY). Cells were maintained in 5% CO_2_ at 37°C during experiments and imaging.

### Fluid Flow Conditions

Two methods were used to induce fluid shear stress over the surface of cells. An oscillatory pump connected to parallel plate flow chambers via hard–walled tubing was used to induce a shear rate of 10 dynes/cm^2^ across the surface of cells plated on glass slides. Additionally, a reservoir was created for the movement of fluid and 5% CO_2_ exchange using hard-walled tubing attached to the outlet of the parallel plate. Static controls were incubated in the same volume of media. Prior to exposure to fluid flow, cells were serum starved (MEM-α, 0.5% FCS) overnight to reduce basal metabolic activity of cells prior to exposure to OFSS. During fluid flow experiments the parallel plate flow chambers and attached hard-walled tubing were placed in an incubator set to 37°C and 5% CO_2_.

Similarly, an orbital shaking platform was used to induce fluid flow over the surface of MC3T3 osteoblasts and MLO-Y4 osteocytes. Cells plated in 6 well culture dishes were placed on an orbital shaking platform within a tissue culture incubator set to 37°C and 5% CO_2_. Prior to experimentation, cells were grown overnight in low-serum α-MEM media supplemented with 0.5% FCS and antibiotics. Cells were subjected to fluid flow generated by 1 mL of media on an orbital platform shaker rotating at a speed of ∼200 rpm (2 Hz) producing a estimated shear rate of ∼10–25 dynes/cm^2^ while inside a tissue culture incubator [10], [38]–[40].

### Src Inhibitor

MC3T3 osteoblasts and MLO-Y4 osteocytes were treated with Src Inhibitor 1 (Santa Cruz Biotechnology, Santa Cruz, CA) (10 µM) for 1 hour before experiments were conducted. Control samples were treated with DMSO.

### Western Blotting Analysis

Cells exposed to static or flow conditions were harvested directly into SDS sample buffer and protein concentrations were determined using amino black method [41]. Equal amounts of protein were loaded onto SDS-PAGE gels for separation and transferred to nitrocellulose. The subsequent primary and secondary antibodies were used: phospho-Src (Y418) (Cell Signaling, Boston, MA), total Src (Cell Signaling, Boston, MA), γ-tubulin (Sigma-Aldrich, St. Louis, MO), lamin B (Santa Cruz, Santa Cruz, CA), HRP conjugated goat anti-rabbit and HRP conjugated goat anti-mouse (Jackson Immunoresearch, West Grove, PA). The secondary antibody signals were detected using a Luminescent Image Analyzer LAS-3000 system (Fujifilm Life Science, Stamford, CT). Densitometry was quantified using Image J software (NIH).

### RNA Extraction, cDNA Synthesis and Quantitative Real-time PCR (qRT-PCR) Analysis

RNA was harvested from cultured MC3T3 osteoblasts and MLO-Y4 osteocytes in Trizol (Invitrogen, Carlsbad, CA). RNA was extracted with chloroform and precipitated with isopropanol. M-MLV reverse transcriptase (Promega, Madison, WI) was used to perform first strand cDNA synthesis. GAPDH (Mm99999915_g1), osteopontin (Mm00436767_m1), and RPLP2 (Mm03059047_gH) real-time PCR primers were obtained (Applied Biosystems, Grand Island, NY). Custom designed primer/probes were prepared for osteocalcin. (forward) 5(-CTGACAAAGCCTTCATGTCCAA-)3 (probe) 5(-AGGAGGGCAATAAGGTAGT-)3 and (reverse) 5(-GGTAGCGCCGGAGTCTGTT-)3. TaqMan Universal PCR Master Mix (Applied Biosystems, Grand Island, NY) was used for amplification in a Mastercycler ep realplex^2^ real-time PCR system (Eppendorf, Westbury, NY). The reaction conditions were as follows: 2 minutes at 50°C; 10 minutes at 95°C; 40 cycles of 15 seconds at 95°C and 1 minute at 60°C. The ΔΔCT method was used to evaluate gene expression between samples. RPLP2 and GAPDH were used as loading control genes.

### Immunofluorescence

Images for all experiments were captured using a Nikon inverted immunofluorescence microscope equipped with a CCD camera. MLO-Y4 osteocytes cells were plated onto coverslips and placed into 6 well culture dishes. Cells were serum starved for 24 hrs (0.5% FCS), subjected to static conditions and fixed immediately or fixed after 20 minutes of OFSS (via orbital shaking platform) with 4% paraformaldehyde solution and permeablilized with 0.2% triton after rinsing with Tris-buffered saline (TBS). Coverslips were treated with normal donkey serum for 30 minutes at 37°C to block non-specific antibody binding. The following primary and secondary antibodies were used: Src and Src Y418 (Cell Signaling, Boston, MA) followed by FITC-conjugated donkey anti-rabbit (Jackson Immunoresearch, West Grove, PA). Texas-Red, phalloidin, and DAPI (Molecular Probes, Eugene, OR) were used for visualizing F-actin and the nucleus, respectively.

Osteoblasts were plated on glass coverslips, ∼2.0×10^5^ cells per slide (∼1.1×10^5^ cells/cm^2^), following 24 hrs of serum starvation (0.5% FCS), cells subjected to static culture conditions were fixed immediately (static) or fixed after OFSS (30 minutes, 1 hour, or 1 hour plus 1 hour) (via oscillatory pump) with 4% paraformaldehyde solution and processed for immunofluorescence by permeabilization with 0.2% triton followed by rinsing in Tris-buffered saline (TBS). Slides were then treated with 1% BSA solution in TBS for 30 minutes at 37°C to block non-specific antibody binding. The following primary and secondary antibodies were used: Pyk2 (BD Biosciences, San Jose, CA), DAPI (Molecular Probes, Eugene, OR), and FITC-conjugated donkey anti-rabbit (Jackson Immunoresearch, West Grove, PA).

### DNA Plasmids

The Src biosensor and Src mutant biosensor were generously obtained from Dr. Yingxiao Wang [42].

### FRET Microscopy

One day prior to imaging, MLO-Y4 cells were electroporated (150 V, 9 ms) with either the Src biosensor or the Src mutant biosensor (10 µg). Cells were plated into 35 mm glass bottom dishes (MatTech, Ashland, MA) and maintained in phenol-free, MEM-α media supplemented with 0.5% FCS and antibiotics. The following day, FRET microscopy was performed using an ISS ALBA FastFLIM system (ISS Inc., Champagne, IL) coupled to an Olympus IX71 microscope equipped with a 60 X/1.2 NA water-immersion objective lens. A 5 mW 448 nm diode laser was modulated by the FastFLIM module of the ALBA system at a fundamental frequency of 20 MHz with up to six sinusoidal harmonics. The modulated 5 mW 448 nm laser was used to excite the donor fluorophore of either the Src biosensor or Src mutant biosensor. The frequency domain FLIM method was used to obtain fluorescent lifetime(s) based on changes in the phase and amplitude of the emission signal compared to the excitation source. The phasor plots, lifetime maps and intensity images were analyzed using ISS VistaVision software (ISS Inc., Champagne, IL). Lifetime maps were generated using a two-component fit for the calculation of the donor lifetime. The phasor plots are used to display the modulation and phase characteristics of the emission signal for every pixel in an image, providing a quantatitive determination of the biosensor probe fluorescence lifetime [43], [44]. Lifetime data from MLO-Y4 cells were extracted from three different regions of interest (ROI): plasma membrane, cytoplasm and nucleus. To ensure lifetime measurements were taken at the appropriate position Bodipy C12 (Invitrogen Corp., Grand Island, NY) (0.01 mg/ml) was used to identify the position of the plasma membrane from the cytosol, as well as mark the outline of the nucleus.

### Nuclear Fractionation

Cells subjected to either static or OFSS treatment in 6 well dishes were washed with phosphate buffered saline, harvested in ice-cold hypotonic buffer (10 mM HEPES, 10 mM KCL, 1.5 mM MgCl_2_, 1 mM DTT and protease inhibitors), passed through a 22-gauge needle five times and were centrifuged for 10 minutes at 13,000 rpm. The cytosolic fraction was saved while the nuclear fraction pellet was resuspended in buffer C (10 mM HEPES, 0.42 M NaCl, 25% Glycerol, 1.5 mM MgCl_2_, 0.5 mD EDTA, ddH_2_O and protease inhibitors) placed on ice and vortexed for 15 seconds every 10 minutes for an hour to swell nuclear proteins out the nuclei. Finally the samples were centrifuged at 4°C for 15 minutes at 14,000 rpm. The clear supernatant was collected and prepared for western blot analysis.

### Immunoprecipitation

For detecting protein-protein interactions *in vivo* co-immunoprecipitation was performed in MC3T3 osteoblasts and MLO-Y4 osteocytes. Immunoprecipitation was performed using Src protein, Src (Y416), MBD2, normal rabbit serum, or normal mouse serum. Immunoprecipitation buffer contained 1% Triton-X-100, 145 mM NaCl, 10 mM Tric-Cl, pH 7.4, 5 mM EDTA, 2 mM EGTA, and 1 mM PMSF. Immune complexes were captured using Protein A sepharose beads (Sigma-Aldrich, Saint Louis, MO) conjugated to either goat-anti rabbit or goat-anti mouse antibody (Jackson Immunoresearch Laboratories, West Grove, PA).

### Statistical Analysis

Statistical significance was assessed by either a two-tailed t-test or a two-way analysis of variance (ANOVA) with a p-value of p<0.05 or less interpreted as statistically significant.

## Results

**Figure 1:**
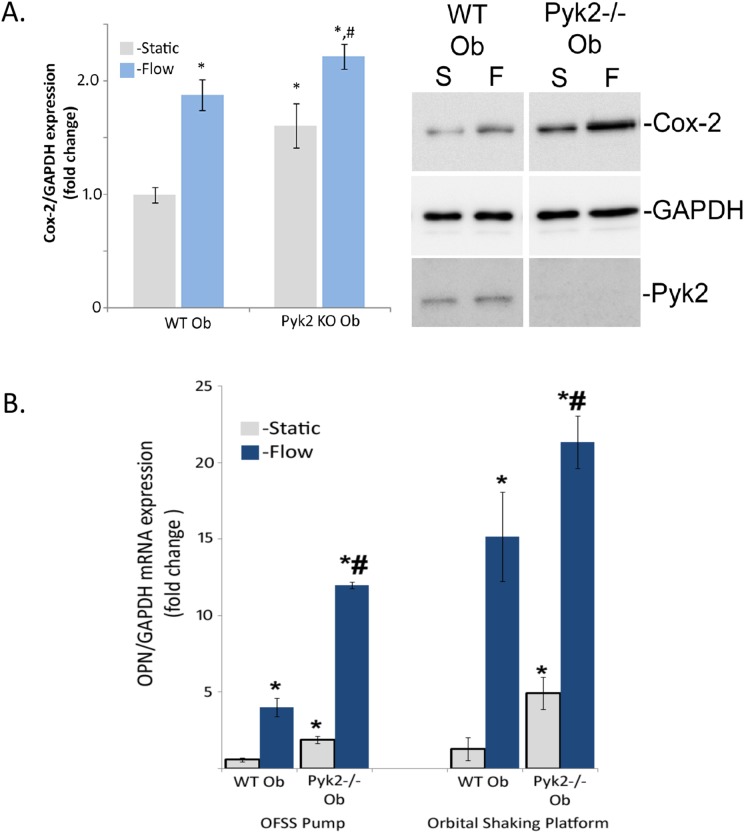
Anabolic protein and gene expression is effected in Pyk2−/− osteoblasts under static and OFSS conditions. (A) Wild-type osteoblasts and Pyk2−/− osteoblasts were exposed to either static of one hour of OFSS. Representative of immunoblots for Cox-2, GAPDH, and Pyk2 show a OFSS-induced increase in Cox-2 protein expression. OFSS does not cause a change in the loading control, GAPDH. Graph represents the densitometry units of Cox-2, normalized to GAPDH. In wild-type osteoblasts, OFSS results in a 1.8 fold change in Cox-2 protein expression. Pyk2−/− osteoblasts have an elevated protein expression of Cox-2 under static conditions, and after OFSS the level of Cox-2 protein expression is highest compared to all other groups (static control, wild-type osteoblasts static control, and wild-type osteoblasts OFSS). Error bars represent standard error. *p<0.05 vs wild-type static control, #p<0.05 vs. wild-type osteoblasts. N = 3 in three separate trials. (B) Wild-type osteoblasts and Pyk2−/− osteoblasts were exposed to either static or 1 hour of OFSS conditions, using either an OFSS pump or orbital shaking platform. Static Pyk2−/− osteoblasts expressed significantly higher levels of osteopontin compared to static wild-type osteoblasts. In response to OFSS, using either method, the absence of Pyk2 further enhances the OFSS-induced osteopontin expression increase. Error bars represent standard error. *p<0.05 vs. wild-type static control, #p<0.05 vs wild-type osteoblasts. N = 3 in three separate trials.

To examine the role of Pyk2 in fluid shear stress-induced expression of anabolic targets of OFSS wild-type osteoblasts and Pyk2−/− osteoblasts were subjected to either static culture or 1 hour of OFSS. OFSS induced a significant 1.8 fold increase in Cox-2 expression in wild-type osteoblasts (Figure 1A). However, in the absence of Pyk2 (Pyk2 KO osteoblasts), Cox-2 expression was significantly elevated compared to wild-type osteoblasts both in static culture and following exposure to OFSS (Figure 1A). OFSS did not alter Pyk2 protein expression in either the wild-type osteoblasts or Pyk2 KO osteoblasts (Figure 1A). To determine if Pyk2 plays a role in regulating other anabolic genes in response to OFSS, relative expression of osteopontin (OPN) mRNA was examined in Pyk2 KO osteoblasts. Under static conditions, Pyk2 KO osteoblasts expressed significantly higher levels of OPN mRNA (Figure 1B). When wild-type osteoblasts and Pyk2 KO osteoblasts were exposed to either static or OFSS conditions using either the OFSS pump or orbital shaking platform, Pyk2 KO osteoblasts expressed enhanced levels of OPN mRNA compared to wild-type osteoblasts in response to OFSS (Figure 1B).

**Figure 2:**
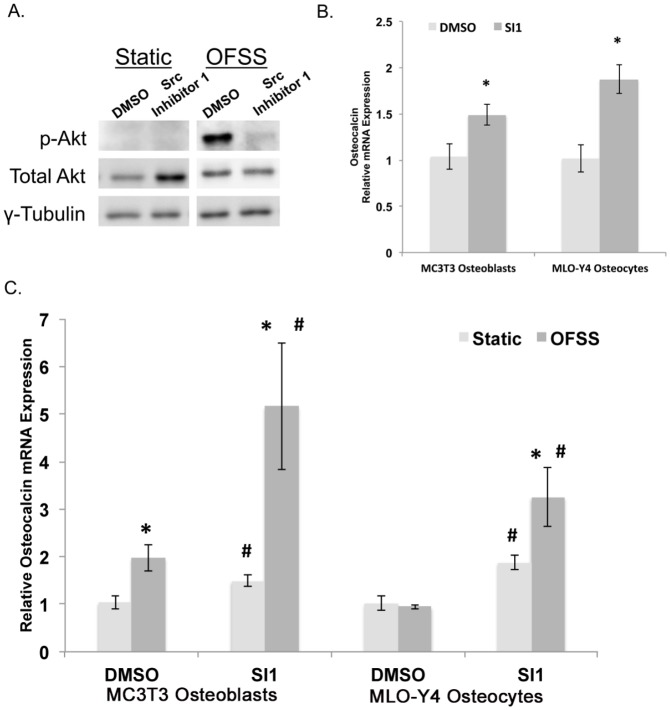
Src kinase represses osteocalcin in static and OFSS conditions. (A) Treating MC3T3 osteoblasts and MLO-Y4 osteocytes with Src Inhibitor 1 (SI1) (10 µM) for 1 hour inhibits Src kinase activity. Western blot analysis of phosphorylated-Akt (Ser308), Total Akt, and γ-tubulin under static and OFSS conditions (1 hour). (B) One hour of SI1 treatment significantly increases the expression of osteocalcin in MC3T3 osteoblasts and MLO-Y4 osteocytes (*p<0.05). (C) OFSS (1 hour) further enhanced the expression of osteocalcin in both MC3T3 osteoblasts and MLO-Y4 osteocytes compared to static controls treated with carrier (DMSO) (p<0.05). *Represents a statically significant increase compared to wild-type static control (p<0.05). #Represents a statically significant difference between treatment groups (#p<0.05) Error bars represent standard error. An n≥3 were used in all experiments and each experiment was repeated at least three times.

Although Src KO osteoblasts were not available, we evaluated the role of Src in expression of osteogenic genes in MC3T3 osteoblasts and MLO-Y4 osteocytes following treatment with a pharmacologic inhibitor of Src activity (Src inhibitor-1, SI1). We first confirmed that SI1 effectively inhibited Src activity by showing that SI1 inhibited fluid flow-induced Akt phosphorylation (Figure 2A). Osteocalcin expression was significantly increased in both MC3T3 osteoblasts and MLO-Y4 osteocytes treated with SI1 under static culture. (Figure 2B). Most importantly, OFSS further enhanced the expression of osteocalcin in both MC3T3 osteoblasts and MLO-Y4 osteocytes treated with SI1 compared to static controls treated with carrier (DMSO) only (2.7 and 3.4 fold change, respectively; Figure 2C). Knocking down Src protein expression via siRNA was attempted, but consistent knockdown of was unsuccessful.

### Pyk2 and Src Accumulate in Perinuclear and Nuclear Regions in Response to OFSS

**Figure 3:**
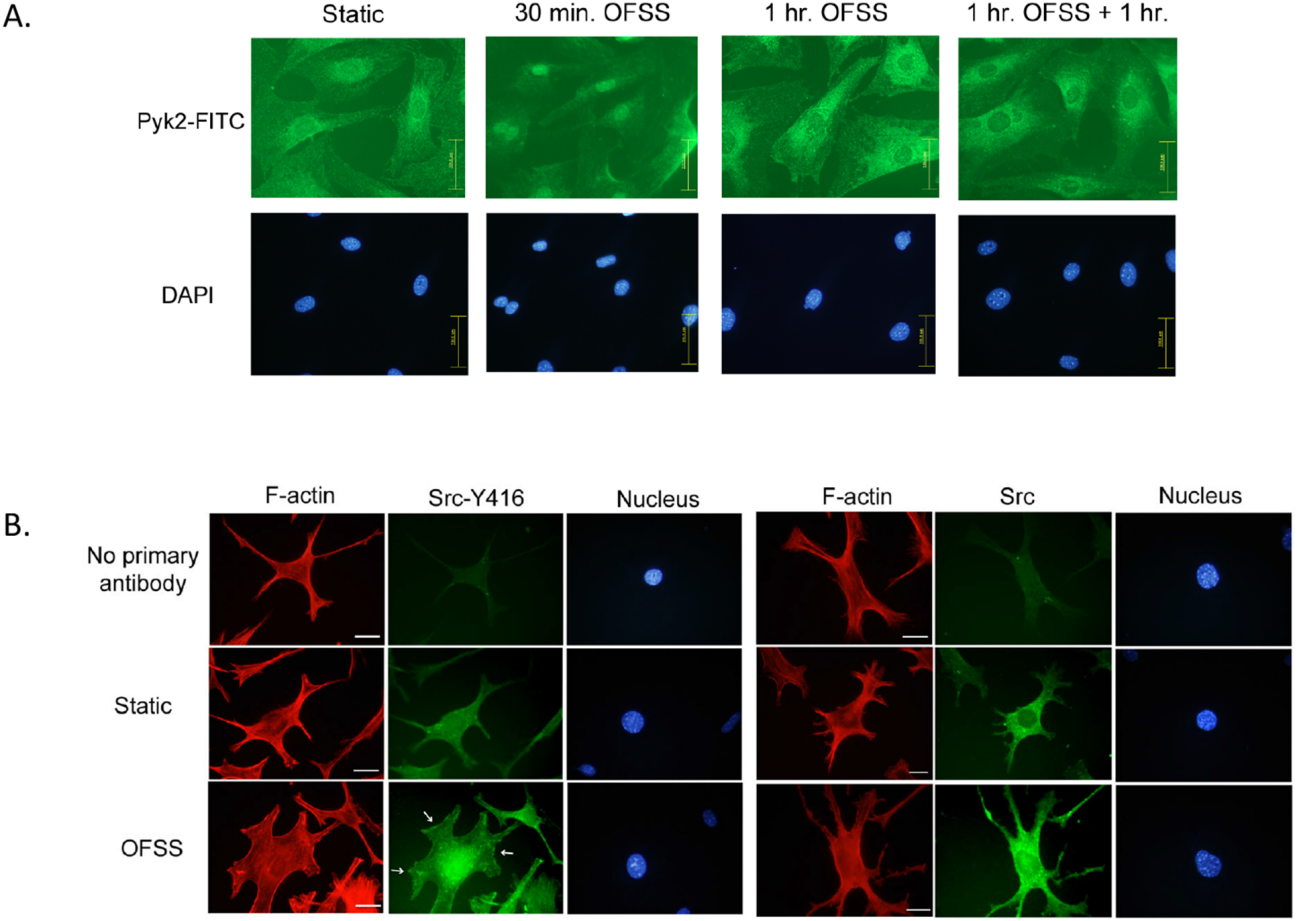
Pyk2 and Src accumulate in perinuclear and nuclear regions in response to OFSS. (A) Immunofluorescence microscopy of osteoblast’s subjected to either static culture conditions or OFSS (30 minutes, 1 hour, or 1 hour+1 hour of rest). Slides were fixed immediately and processed for immunofluorescence using antibodies against Pyk2, followed by FITC-conjugated secondary antibodies. The nucleus was visualized using DAPI. Scale bars = 100 µm (B) OFSS induces accumulation of Src at perinuclear/nuclear regions in MLO-Y4 osteocytes. Immunofluorescence microscopy of MLO-Y4 osteocytes subjected to static culture conditions or OFSS for 20 minutes. Slides were fixed immediately and processed for immunofluorescence using antibodies against activated Src (Y416 followed by FITC-conjugated secondary antibodies). F-actin was visualized using Texas-Red Phalloidin and the nucleus was visualized using DAPI. White arrows indicate focal adhesions. Scale bars = 25 µm (B) Immunofluorescence microscopy of MLO-Y4 osteocytes subjected to static culture conditions or OFSS for 20 minutes. Slides were fixed immediately and processed for immunofluorescence using antibodies against total Src followed by FITC-conjugated secondary antibodies. F-actin was visualized using Texas-Red Phalloidin and the nucleus was visualized using DAPI. Scale bars = 25 µm.

The localization pattern of both Pyk2 and Src in response to OFSS was analyzed using immunofluorescence microscopy. The cellular distribution of Pyk2 in osteoblasts was observed under static and OFSS (30 minutes, 1 hour, or 1 hour+1 hour of rest) conditions. Static osteoblasts displayed a relatively even distribution of Pyk2 throughout the cells, with a subtle concentration of Pyk2 in the perinuclear/nuclear region (Figure 3A). We found that Pyk2 preferentially accumulated in the nuclei following exposure to 30 minutes of OFSS. After exposure to one hour of OFSS, the accumulation of Pyk2 in the nucleus was less robust than at the 30 minute OFSS time point (Figure 3A). In response to 1 hour of OFSS and 1 hour of rest, Pyk2’s absence in the nucleus was even further enhanced compared to static. Comparable results were observed in MLO-Y4 osteocytes (not shown). These results suggest that Pyk2 is capable of shuttling between the cytoplasm and the nucleus in response to OFSS.

We next examined changes in the distribution of Src in response to OFSS. Immunofluorescence microscopy indicated a shift in the distribution of both total Src and activated Src (as assessed by phosphorylation at tyrosine residue Y416) following as little as 20 minutes of exposure to OFSS in MLO-Y4 osteocytes. Using an antibody that recognizes only activated Src phosphorylated at tyrosine 416 (Y416) we found that activated Src accumulated in the perinuclear/nuclear area of MLO-Y4 osteocytes after exposure to OFSS (Figure 3B). Total Src protein also increased modestly in the perinuclear/nuclear regions after OFSS, but was not as pronounced as that of the activated (Y416) Src (Figure 3B). These results suggest that Src activation (as measured by phosphorylation at Y416) may be required for Src to accumulate in the nucleus of the cell. We saw no evidence that Src was shuttling back out of the nucleus during the time course of these experiments.

### Nuclear Src Activity Increases in Response to OFSS

To examine the observation of increased perinuclear/nuclear Src accumulation in response to OFSS we used live cell imaging to monitor changes in Src activity in response to OFSS. To directly examine changes in the sub-cellular distribution of Src tyrosine kinase activity in MLO-Y4 osteocytes in response to OFSS we utilized a Src biosensor to measure Src activity by FRET microscopy. The changing FRET signal from the Src biosensor probe was detected using fluorescent lifetime imaging microscopy (FLIM), which measures the shortened donor lifetime that results from FRET. This approach allows us to map with pixel level resolution the sub-cellular locations of changing Src protein activity. The Src biosensor used here is in a closed conformation under conditions of low endogenous Src activity, resulting in high FRET efficiency and a shortened donor lifetime. Upon phosphorylation of the substrate peptide, a sequence derived from p130cas, by endogenous Src, the substrate binds to the phosphopeptide-binding pocket of the SH2 domain, resulting in a more open conformation and diminished FRET leading to an increased donor lifetime. Thus, an increase in Src biosensor lifetime indicates an increase in Src kinase activity.

**Figure 4:**
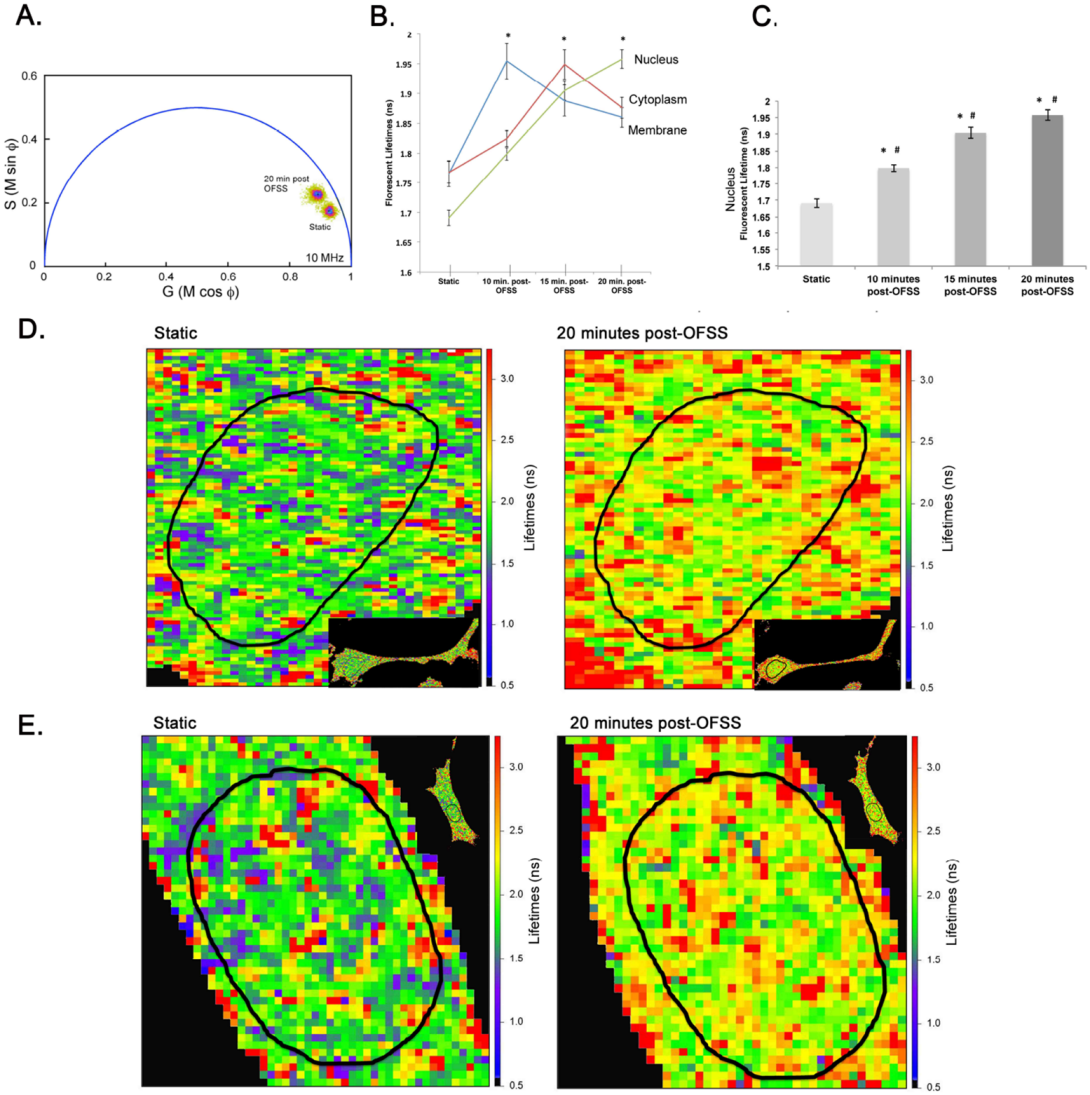
OFSS causes an increase in nuclear Src activity. (A) Composite phasor plot analysis of static Src biosensor lifetime and 20 minutes post-OFSS Src biosensor lifetime. (B) Graph represents n = 3 in which the average lifetime of each ROI (30) at each time point analyzed. Error bars represent standard error. (C) Graph of nuclear Src lifetimes. *Represents a statically significant increase compared to static control (*p<0.001). #Represents a statically significant difference between other OFSS time points examined (#p<0.05) Error bars represent standard error. (D, E) Lifetime maps of the same MLO-Y4 osteocyte under static, 10 minutes, 15 minutes or 20 minutes post-OFSS.

The phasor plot analysis comparing the total population of Src biosensor in MLO-Y4 cells prior to or following exposure to OFSS clearly showed a shift towards longer lifetimes of the Src biosensor, indicating a global increase in Src activity throughout MLO-Y4 cells in response to OFSS (Figure 4A). Following exposure to 5 minutes of OFSS, lifetimes of the Src biosensor donor fluorophore were determined for three distinct sub-cellular compartments (regions of interest, ROI) – the membrane, cytoplasm and nucleus - at 10, 15 and 20 minutes post-OFSS using FLIM analysis. Prior to OFSS, Src activity in the nucleus was significantly lower than at either the membrane or in the cytoplasm (Figure 4B). Most importantly, a significant increase in nuclear Src activity was seen at each time point following OFSS and increased steadily during the 20 min post-OFSS period (Figure 4C). Lifetime maps of the nucleus under static and post-OFSS conditions illustrate the increase in Src activity in response to OFSS (Figure 4D). In contrast, Src activity at the membrane and in the cytoplasm peaked at 10 and 15 minutes post-OFSS, respectively, and then decreased at 20 minutes post-OFSS (Figure 4B). Prior to OFSS, the average lifetime of the Src biosensor in the nucleus was 1.69±0.01 nanoseconds. At 10, 15 and 20 minutes post-OFSS nuclear Src biosensor lifetimes significantly increased compared to static (1.80±0.01, 1.90±0.02, and 1.95±0.02 nanoseconds, respectively) (Figure 4B).

In sharp contrast, there was no change in the fluorescent lifetime of a mutant Src biosensor (Y662F Y664F) following 5 minutes of OFSS (Figure S1). The mutation of the tyrosine residues in the p130cas substrate blocks Src tyrosine kinase activation of the biosensor, and also changes its conformation, resulting in higher basal lifetimes compared to the Src biosensor.

### Src Activation Increases in the Nucleus in Response to OFSS

**Figure 5:**
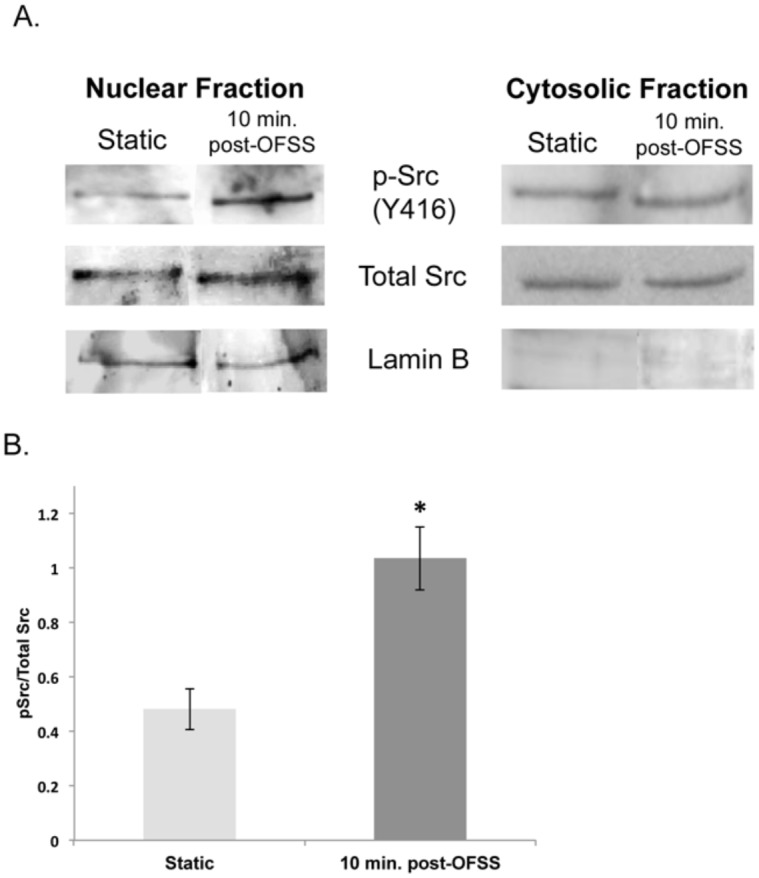
Src activation increases in the nucleus in response to OFSS. (A) Western blot analysis of nuclear fractionation blotted for Src activation (Y416), total Src and lamin B in MLO-Y4 osteocytes exposed to 5 minutes of OFSS or static culture conditions. (B) Graph represents quantification of Src activation (Y416)/total Src in nuclear fractions. Error bars represent standard error. Statistically significant difference between static and 10 minutes post-OFSS (*p<0.05). An n≥3 were used in the repeated experiments.

To validate the observation of increased Src activity in the nucleus of MLO-Y4 cells in response to OFSS; nuclear fractionation followed by Western blot analysis was performed. MLO-Y4 osteocytes were exposed to either static conditions (control) or 5 minutes of OFSS. The control cells or OFSS cells were collected 10 minutes-post-OSS. Src activation, as measured by phosphorylation at Y416, was performed by Western blot. In response to 5 minutes of OFSS activated Src in the nucleus was increased at 10 minutes post-OFSS compared to static conditions (Figure 5).

### Pyk2 and MBD2 Complex under Basal Conditions and Increase their Association in Response to OFSS

**Figure 6:**
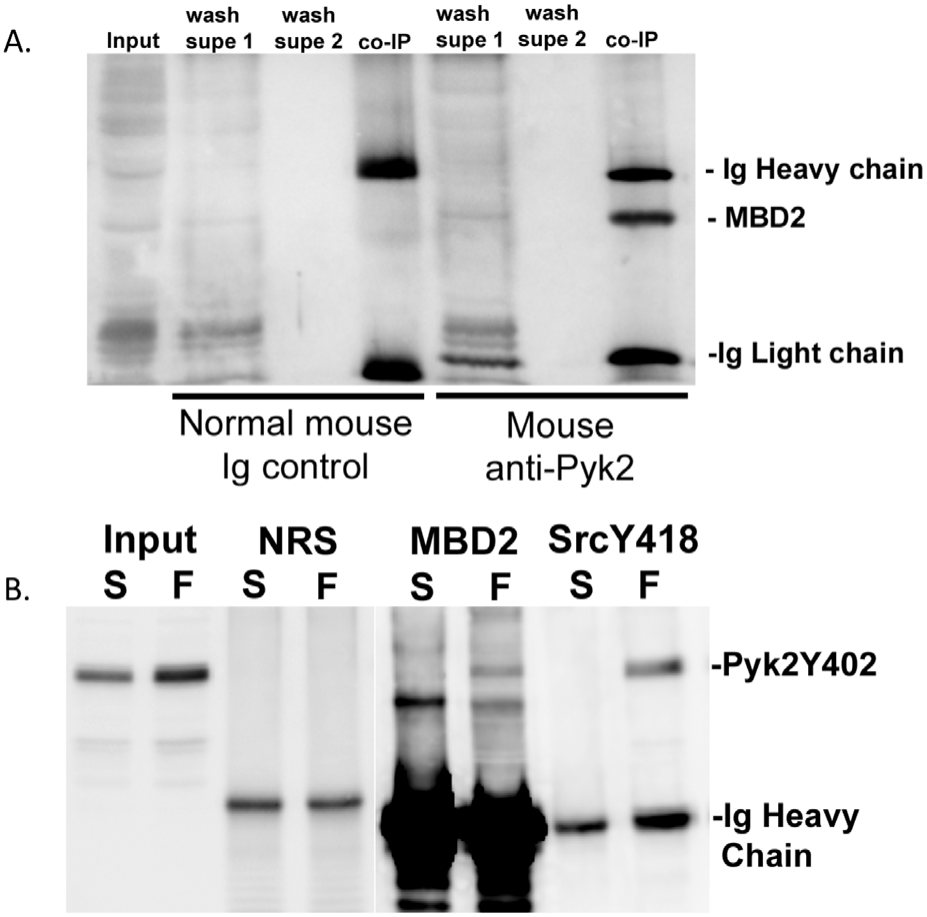
A complex between MBD2 and Pyk2 forms under static conditions in MLO-Y4 osteocytes, while OFSS-induces the association of MBD2 and Pyk2 (Y402) and Src (Y418) and Pyk2 (Y402) in MLO-Y4 osteocytes. (A) Co-immunoprecipitation between MBD2 and Pyk2 was performed from MLO-Y4 osteocytes harvested under static conditions. MBD2 was not associated with Pyk2 when control normal mouse Ig was used in the immunoprecipitation. Anti-MBD2 antibody was used to probe the blot. (B) Co-immunoprecipitation was performed in MLO-Y4 osteocytes harvested under static (S) or OFSS (F) conditions. Normal rat serum (NRS), MBD2, and Src (Y418) were the antibodies used for the immunoprecipitation. Anti-Pyk2 (Y402) specific antibody was used to probe the blot.

To examine the functional significance of Pyk2 translocation to the nucleus in response to OFSS we evaluated Pyk2’s association with MBD2. In muscle cells the interaction of FAK, a closely related family member to Pyk2, and MBD2 in the nucleus has been observed during differentiation, leading to the disruption of the repression complex and increased expression of myogenin [45]. Therefore, we performed a co-immunoprecipation assay to examine the possibility that OFSS was inducing the formation of a Pyk2-MBD2 complex. Antibodies to either Pyk2 or MBD2 were used to detect co-immunoprecipitated proteins by reciprocal western blot analysis. In osteocyte lysates from cells maintained in static culture, endogenous Pyk2 formed a complex with MBD2 (Figure 6A). A complex of Pyk2/MBD2 was detected using antibodies to either Pyk2 or MBD2. Next, we compared the association of Pyk2/MBD2 under static and OFSS conditions using antibodies that are specific for the activated form of Pyk2 (Y402). Under static culture conditions no association between MBD2 and Pyk2 was detected. However, in response to 20 minutes of OFSS, MBD2 was detected in the Pyk2 IP complex suggesting that MBD2 may interact with activated Pyk2 that is phosphorylated at tyrosine 402 (Figure 6B) in response to OFSS. Interestingly, OFSS also induced an association between Pyk2 (Y402) and Src (Y418) suggesting the formation of a Pyk2-Src-MBD2 complex in the nucleus (Figure 6B).

## Discussion

We suggest that Pyk2 and Src may serve as an “off switch” to suppress the anabolic response of bone subjected to mechanical load. We have previously proposed the mechanosomes model to describe how mechanical stimuli sensed at the plasma membrane, results in changes of gene transcription [46]–[48]. A mechanosome consists of an adhesion-associated protein and a nucleocytoplasmic shuttling transcription factor. There are two forms of mechanosomes, a “GO” mechanosome and a “STOP” mechanosome. A “GO” mechanosome functions to promote the anabolic response of bone to mechanical loading, while a “STOP” mechanosome functions to suppress the anabolic response of bone to loading. Our findings suggest Pyk2 and Src have the capacity to function as a “STOP” mechanosome. Once activated by mechanical stimulation, Pyk2 and Src change their pattern of localization and serve as a nuclear mechanism to prevent an over-reaction to physical stimulation.

The aim of this study was to better understand the signaling mechanisms that osteoblasts and osteocytes use to suppress the anabolic response of bone to mechanical loading. Src and Pyk2 were examined due to their shared knockout phenotype, increased bone mass. In the absence of Pyk2, Cox-2 and osteopontin expression are modestly increased. OFSS further enhanced Cox-2 and osteopontin expression in Pyk2 null osteoblasts. Therefore, Pyk2 functions to suppress the expression of the anabolic bone gene osteopontin, as well as the expression of Cox-2 protein under static and OFSS conditions. Immunofluorescence microscopy was performed to evaluate if OFSS induced changes in the subcellular localization of Pyk2. Osteoblasts under static conditions displayed a relatively even distribution of Pyk2 throughout the cytoplasm, with a subtle concentration of Pyk2 in the perinuclear/nuclear region (Figure 3A). Evidence that Pyk2 was capable of shuttling into and out of the nucleus in response to OFSS was seen by an initial accumulation of Pyk2 in the nucleus (after 30 minutes of OFSS) followed by an absence in the nucleus (following 1 hour of OFSS and 1 hour of rest). An OFSS-induced nucleocytoplasmic shuttling mechanism for Pyk2 has yet to be described, but other studies in fibroblasts have observed Pyk2’s nucleocytoplasmic shuttling behavior in response to membrane depolarization [49],[50]. Nucleocytoplasmic shuttling of Pyk2 is possible through the nuclear localization sequence (NLS) and the nuclear export sequence (NES) that are both located in the FERM domain [51]–[53]. The changes observed in Cox-2 expression and osteopontin transcription might be mediated by an OFSS-induced association of Pyk2 and MBD2. In MLO-Y4 osteocytes a basal interaction between Pyk2 and MBD2 was observed. When examining the activated form of Pyk2 (Y402), an increase in its association with MDB2 occurs in response to 20 minutes of OFSS. While not a transcription factor, MBD2 is a member of the methyl CpG-binding protein family and functions to repress transcription [54]–[56]. Specifically, MBD2 and methyl CpG binding protein 2 (MeCP2) bind heterochromatin through their interaction with methylated DNA at CpG islands. The complex then translates the DNA methylation signal into transcriptional repression by recruiting histone deacetylases and other silencing complexes to sustain a heterochromatic state [57], [58]. In muscle cells an interaction between FAK and MBD2 in the nucleus has been observed during differentiation, leading to the disruption of the repression complex and increased expression of myogenin [45]. Similarly, in response to membrane depolarization Pyk2 binds MBD2 in the nucleus of nerve cells, but the functional outcome of this observation has yet to be explained [49]. These findings suggest the possibility that MBD2 might be a component of a mechanosome containing Pyk2 and/or Src kinase.

A second aspect of this study was to investigate a potential role for Src tyrosine kinase in transcriptional regulation of anabolic gene expression in MC3T3 osteoblasts and MLO-Y4 osteocytes, principally in response to OFSS. Expression of osteocalcin, a marker of bone formation *in vivo*, was increased when Src activity was inhibited in MC3T3 osteoblasts and MLO-Y4 osteocytes. Furthermore, we show for the first time that the increase in osteocalcin expression normally induced by OFSS was further enhanced in MC3T3 osteoblasts and MLO-Y4 osteocytes when Src activity was inhibited by treatment with SI1 (compared to control cells in which Src activity was not inhibited). Thus, our results suggest Src normally functions to suppress osteocalcin expression under conditions of both static culture and OFSS. Osteocalcin is an important protein associated with bone formation and its levels in serum directly correlate with measurements of bone mineral density [59], [60]. Osteocalcin expression is increased in response mechanical loading in both *in vivo* and *in vitro* models [61], [62]. We also analyzed expression of cyclooxygenase-2, runx2, osterix, and osteopontin and found inhibition of Src had no impact on OFSS-induced increases in their expression (data not shown). Our data suggests that while Src affects the basal expression of RunX2, osterix and osteopontin, as previously reported, OFSS-induced changes in osteocalcin expression are Src independent. We used a Src biosensor to demonstrate that Src activity increased in the nucleus of MLO-Y4 osteocytes in response to OFSS; this was further confirmed using nuclear fractionation. Together these results support the novel concept that Src plays a role in osteoblastogenesis by functioning to curb the anabolic response of MC3T3 osteoblasts and MLO-Y4 osteocytes to OFSS via a mechanism that involves a mechanically-induced increase in nuclear Src activity.

Taken together our immunolocalization, FRET microscopy and nuclear fractionation studies suggest that exposure of MLO-Y4 cells to OFSS results in an increase in the amount of activated Src (Y416 phosphorylation) in the perinuclear/nuclear regions and an increase in Src tyrosine kinase activity in the nucleus in response to OFSS. A subtle increase in total Src in the perinulear/nuclear region was seen in response to OFSS. In contrast, a more pronounced increase in activated Src (Y416) was seen in the perinuclear and/or nuclear regions of MLO-Y4 osteocytes (Figure 3B) suggesting that the population of Src that accumulates in this region is activated (as assessed by Y416 phosphorylation). Our nuclear fractionation and FRET data directly demonstrate that Src tyrosine kinase activity increased in the nucleus in response to OFSS. This novel result suggests a previously unrecognized role for Src tyrosine kinase activity in regulating the transcriptional response of MLO-Y4 osteocytes to OFSS. There is not a widely accepted mechanism to explain how Src translocates into the nucleus, however myrstoylation has been suggested to function in regulating transport of Src to the nucleus [63].

Further studies will be needed to determine the detailed molecular mechanism(s) by which a Pyk2 and Src complex and its nuclear binding have the capacity to directly alter transcription and the epigenome.

## Supporting Information

Figure S1 **Mutant Src biosensor does not respond xxto OFSS in MLO-Y4 osteocytes.** (A) Phasor plot overlay of static mutant Src biosensor lifetime and mutant Src biosensor lifetime exposed to OFSS. (B) Lifetime maps of the same MLO-Y4 osteocyte at static and 20 minute post-OFSS time points.
(TIF)
