# Supplementary figures and images for: Mechanical Loading in Osteocytes Induces Formation of a Src/Pyk2/MBD2 Complex That Suppresses Anabolic Gene Expression
Source: PLoS One. 2014 May 19;9(5):e97942. doi: 10.1371/journal.pone.0097942 (PMC4026426; doi:10.1371/journal.pone.0097942)

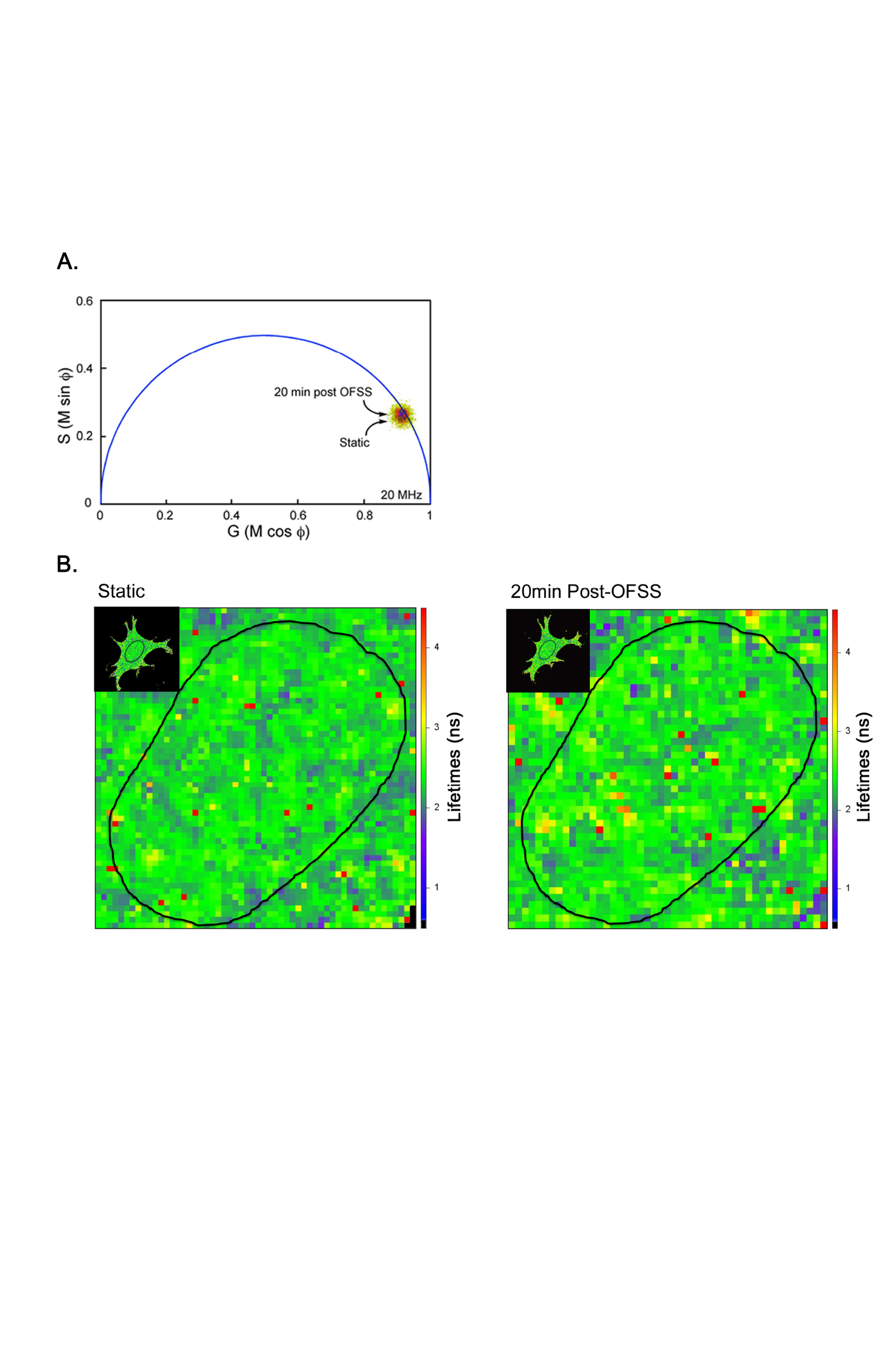

Supplement: Figure S1 — Mutant Src biosensor does not respond xxto OFSS in MLO-Y4 osteocytes. (A) Phasor plot overlay of static mutant Src biosensor lifetime and mutant Src biosensor lifetime exposed to OFSS. (B) Lifetime maps of the same MLO-Y4 osteocyte at static and 20 minute post-OFSS time points. (TIF) [file pone.0097942.s001.tif]
